# Machine Learning Techniques for Effective Pathogen Detection Based on Resonant Biosensors

**DOI:** 10.3390/bios13090860

**Published:** 2023-08-31

**Authors:** Guoguang Rong, Yankun Xu, Mohamad Sawan

**Affiliations:** CenBRAIN Neurotech Center of Excellence, School of Engineering, Westlake University, 600 Dunyu Road, Xihu District, Hangzhou 310030, China; rongguoguang@westlake.edu.cn (G.R.); xuyankun@westlake.edu.cn (Y.X.)

**Keywords:** machine learning, support vector machine, multilayer perceptron, photonic biosensor, signal processing, Tamm plasmon polariton, localized surface plasmon resonance

## Abstract

We describe a machine learning (ML) approach to processing the signals collected from a COVID-19 optical-based detector. Multilayer perceptron (MLP) and support vector machine (SVM) were used to process both the raw data and the feature engineering data, and high performance for the qualitative detection of the SARS-CoV-2 virus with concentration down to 1 TCID_50_/mL was achieved. Valid detection experiments contained 486 negative and 108 positive samples, and control experiments, in which biosensors without antibody functionalization were used to detect SARS-CoV-2, contained 36 negative samples and 732 positive samples. The data distribution patterns of the valid and control detection dataset, based on T-distributed stochastic neighbor embedding (t-SNE), were used to study the distinguishability between positive and negative samples and explain the ML prediction performance. This work demonstrates that ML can be a generalized effective approach to process the signals and the datasets of biosensors dependent on resonant modes as biosensing mechanism.

## 1. Introduction

The global COVID-19 pandemic has had a huge impact on the world’s health and economy [1]. The fast-spreading virus SARS-CoV-2 virus is the main culprit of this phenomenon, and detection of the virus in human populations is crucial for curbing the pandemic [2]. Traditional detection approaches include the nucleic acid amplification test (NAAT) [3] and antigen detection [4] techniques. Currently, the mainstream is quantitative polymerase chain reaction (qPCR) [5], which is a kind of NAAT that has high sensitivity and specificity, but requires a clean environment, bulky and expensive equipment, and trained personnel. Therefore, qPCR is not suitable for onsite, fast turnaround detection or for population-scale screening, which are often required in pandemic control scenarios [6]. To complement qPCR tests, antigen detection based on lateral flow [7] has also been employed in both home use and self-testing. However, antigen detection is limited in detection sensitivity and specificity, hindering its efficacy in fighting a pandemic [8]. There is still a lack of rapid, accurate, and low-cost detection techniques that can be deployed onsite for population-scale epidemic screening and/or surveillance [9], especially for regions with limited resources [10].

Biosensors have been proposed for the detection of SARS-CoV-2 [11]. Biosensor technologies have high sensitivity, good specificity, fast turnaround, ease of operation, low cost, and onsite deployment capability [12,13]. We have previously proposed a photonic biosensor with high sensitivity and specificity for the fast, on-site detection of SARS-CoV-2 [14,15]. The biosensor is based on a nanoporous silicon material fabricated via a CMOS-compatible silicon process and nanophotonic working principles of localized surface plasmon resonance (LSPR) [16] and Tamm plasmon polariton (TPP) [17,18]. The measurement of the biosensor is based on reflection spectroscopy [14].

We also developed handheld and high-throughput detection systems [19] that can collect the reflection spectrum of biosensors and process the spectral data to determine the detection results efficiently. The high-throughput detection system is suitable for a population-scale screening of infection, and the handheld detection system is for home use or self-tests. The spectral data processing algorithm works by recognizing the characteristic resonant valleys in the reflection spectrum of the biosensor and determines the detection results by judging if there is spectral red shift in the characteristic resonant valleys. This is the often-used and so-called “find peaks” technique, with its name originating from the MATLAB function findpeaks(). This technique can also be implemented on field programmable gate arrays (FPGAs) for fast and efficient processing of signals from an array of biosensors [20]. In addition, researchers have proposed the interferogram average over wavelength (IAW) technique to process the signals of optical biosensors that depend on a spectral shift in the characteristic resonant features, which can achieve sensitivity enhancement compared with spectral shift detection [21]. Detection of changes in reflection intensity due to a shift in spectral features in the spectrum has also been used to detect biomolecules in real time [22]. However, both IAW and light-intensity measurement techniques are subject to spectral amplitude fluctuations and thus require highly stable spectroscopy systems, such as a stable light source and high signal-to-noise ratio spectrometers.

In this work, we demonstrate that it is advantageous to utilize artificial intelligence technology, more specifically machine learning (ML) algorithms, to process the spectral data of the biosensor [23]. Instead of depending on programming, its algorithm is learnt from a big volume of data [24]. Machine learning has been used for computer vision [25], face recognition [26], autonomous driving [27,28], auxiliary decision making [29,30], brain–machine interface [31], cancer diagnosis and assessment [32], and chess game [33]. It includes supervised learning, unsupervised learning, and reinforcement learning [34]. Supervised learning (SL) is an algorithm that learns from massive, labeled datasets and generates prediction models that can work to generate labels for new datasets. SL includes support vector machine (SVM) [35], multilayer perceptron (MLP) [36], linear regression [37,38], linear discriminant analysis [39,40], K-nearest neighbor [41,42], decision tree [43,44], and naïve Bayes [45,46]. In this work, we demonstrate that SVM and MLP can be used for processing of the photonic biosensor signal and dataset. Compared with previously proposed techniques, the ML technique has the following advantages: (1) there is no need to find the appropriate parameters of the algorithm, e.g., the findpeaks() function, in a trial-and-error way to guarantee accurate recognition of spectral features; (2) there is no need to discriminate between redshift or blueshift, which can be an extra issue in algorithm design; (3) it is not sensitive to spectral amplitude fluctuations, so the requirements for stable and expensive hardware are relaxed; (4) it is generalizable to all kinds of sensors with salient features in the response signal, which serve as the basis for discriminating between positive and negative responses.

Data visualization approaches can help us to understand the distribution of the dataset and discover the distinguishability of the dataset. T-distributed stochastic neighbor embedding (t-SNE) is a prevalent approach to map high-dimensional data to low-dimensional embedding [47]. In this contribution, we also implemented the t-SNE approach on a SARS-CoV-2 detection dataset to clarify the distinguishability of the biosensor dataset so that a better understanding of the data processing and ML prediction performances could be obtained.

## 2. Materials and Methods

### 2.1. Biosensor Working Principal and Measurement Setup

**Figure 1:**
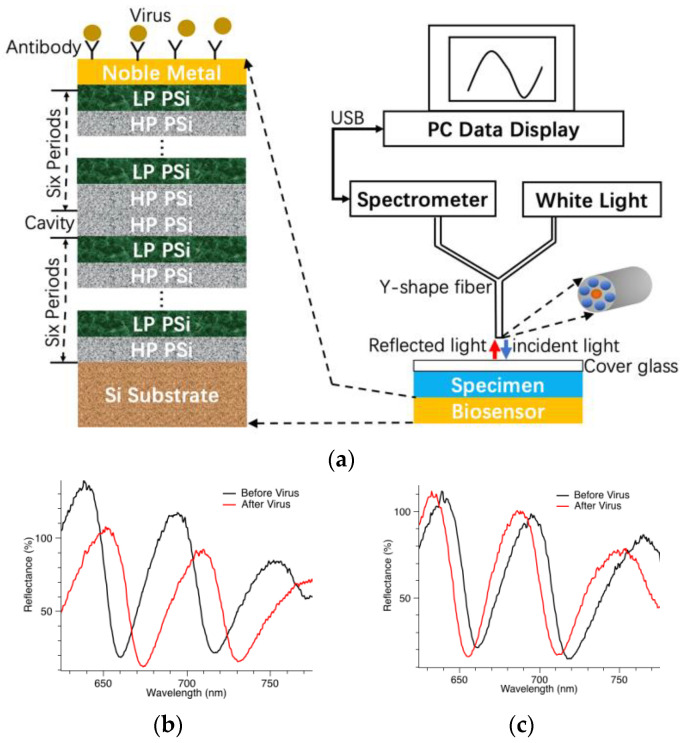
Photonic biosensor: (**a**) structure and its reflection spectroscopy measurement; (**b**) typical example of redshift showing resonant valleys in reflection spectra; (**c**) blueshift of resonant valleys in reflection spectra.

As shown in Figure 1a, the biosensor is basically a porous silicon microcavity consisting of two Bragg reflectors and one resonant cavity [14,48].

One Bragg reflector is six periods of alternating low porosity (LP) and high porosity (HP) porous silicon (PSi) thin films of thickness equal to a quarter resonant wavelength. Noble metal thin film is deposited on top of the porous silicon. Because of the nanoporous structure of the porous silicon material, the conformally deposited noble metal thin film is also porous. When light is incident on the surface of the biosensor, some of its energy is coupled into localized surface plasmon resonance (LSPR) [48] supported by the nanostructures of the noble metal thin film. In addition, some of its energy also couples into the Tamm plasmon polariton (TPP) supported by the interface between the top Bragg reflector and the noble metal thin film [49]. Therefore, the LSPR and TPP are simultaneously excited by the incident light and couple with each other, forming a strong field confinement around the noble metal thin film. If specific antibodies are immobilized beforehand on the surface of the noble metal, they can capture the SARS-CoV-2 virus specifically. Such binding events cause an addition of biomaterials around the noble metal thin film, and the added biomaterial interacts strongly with the coupled LSPR and TPP field. This is the working mechanism of the biosensor for the sensitive detection of the virus. As shown in Figure 1a, in order to measure the signals of the biosensor, reflection spectroscopy is used. A white light source provides the incident light, which passes through the Y-shape fiber and shines vertically onto the biosensor surface. The light reflected from the biosensor surface is collected by the Y-shape fiber and passes into the spectrometer for data analysis. The Y-shape fiber consists of six circumferential fibers guiding incident light, and one central fiber guiding reflected light.

Figure 1b,c show the representative reflection spectra of the biosensor. They have characteristic resonant valleys that are in the spectral range of 600–800 nm in wavelength. If there are viruses binding with antibodies on the biosensor surface, the binding events cause a shift in the spectral features to a longer wavelength, which is called “redshift”. For example, Figure 1b shows such a case where the virus binds with antibodies, redshift occurs, and the detection result is determined to be positive. On the other hand, if there is no virus binding with antibodies on the biosensor surface, there is no shift in the spectral features, i.e., almost overlapping spectra for both before and after binding reaction. In the third case, there could also appear a shift in the resonant features to a shorter wavelength, which is also called “blueshift”. In such cases, the detection result is determined to be negative. Figure 1c shows an example of blueshift. In summary, the principle of the biosensor is based on interactions between biomaterials and photonic energy, and the detection result is determined based on a shift in the spectral features in the optical spectrum collected from the reflection spectroscopy measurement.

### 2.2. Data Preprocessing

This dataset was obtained from detection experiments of inactivated SARS-CoV-2 in clinical swab specimens, with virus concentrations as low as 1 TCID_50_/mL [14]. Figure 1 shows example spectra of the biosensor for positive and negative detection results. For the positive result, there is spectral redshift; and for the negative result, there is either no spectral shift or there is spectral blueshift. The experimental data were collected via reflection spectroscopy with the corresponding spectra for before and after applying specimens on the biosensor surface. Each spectral data contained 2048 data points representing reflection intensities, with a data-to-data spacing of 0.48 nm in the wavelength range of 200–1200 nm. We usually needed to carry out preprocessing of the spectral data before the data analysis, which included normalization and artifacts removal. Furthermore, normalization was implemented on each of the data samples for the purpose of training convenience. Spectral data of both before and after adding specimens were combined as a single sample, so that the size of the reformed sample was 2 × 2048, or 4096. Each detection experiment was regarded as a sample for either training or testing purposes. After several outliers were removed to clean the dataset, there were 486 negative samples and 108 positive samples left in total for the classification model training and prediction test.

### 2.3. Feature Engineering

**Figure 2:**
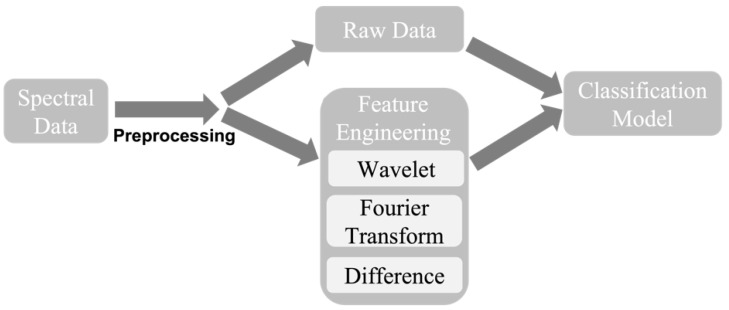
Simplified block diagram of the data-processing procedure. Raw data and feature engineering methods were used in the experiments.

As shown in Figure 2, the input to the model was 4096 data. This required 4096 input neuron nodes, which could be a computational burden. In addition to this raw data approach, the input could also contain features extracted from the data. We propose feature engineering methods comprising three different approaches—wavelet transform, Fourier transform, and spectral difference. For the wavelet domain, we used the wavelet transform with scales of 30 and took the average of each scale, which generated 30 features for each spectral curve. Two curves (before and after virus) generated 60 wavelet-based features. In terms of the Fourier domain, we found that most information appeared in the low-frequency range (<50 Hz), so that we took the average of each 5 Hz in order from 0 to 50 Hz, so that 10 features for each spectral curve and 20 features for spectra pairs were obtained in the Fourier domain. For the spectral difference, we utilized the difference between the spectral data before and after the binding reaction on the biosensor, instead of two separate spectra. There were three features selected from the spectral difference: mean, variance, and sign change rate.

Eventually, for each training sample containing spectral data of before and after the reaction, the wavelet transform and Fourier transform domain features needed the spectra of both before and after the reaction, and spectral difference features only needed the difference between the spectra before and after the reaction. Therefore, there were 83 (60 wavelet domain + 20 Fourier domain + 3 spectral difference) features selected for the classification experiments.

### 2.4. Classification Models

**Figure 3:**
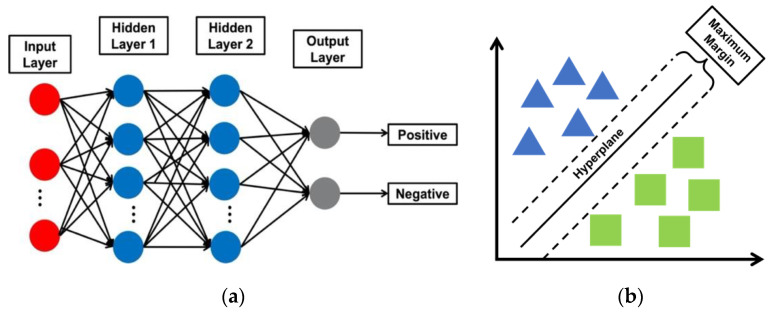
(**a**) Simplified diagram for illustration of MLP architecture; (**b**) SVM explanation.

All the samples were randomly shuffled and separated as 70% for training and 30% for testing. This allocation ratio is a practical standard for benchmark performance. Multilayer perceptron (MLP) and support vector machine (SVM) models were used since they are usually considered as efficient ML models capable of achieving baseline performance. As shown in Figure 3, in terms of the MLP model, two hidden layers with 100 and 50 neuron nodes with a sigmoid activation function were implemented, with the optimizer as a stochastic gradient decent solver, and the learning rate and epoch set as 0.1 and 30, respectively. The number of layers and the number of neurons in each layer were optimized through N ablation study, in which different numbers of layers and different pairs of the number of neurons were tested. Finally, we found that the two layers with 100 and 50 neurons were expected to be the best in final performance and learnable parameters. Decreasing the number of neurons decreased the prediction accuracy slightly. As for the SVM model, we set the gamma parameter of the radial basis kernel function as 1.

### 2.5. Control Experiments

For the control experiments, we detected SARS-CoV-2 specimens with photonic biosensors, which did not have specific antibodies immobilized on the biosensor surface beforehand. There were a total of 732 data samples for detecting SARS-CoV-2 virus specimens of various concentrations, and 36 data samples for detecting specimens containing no SARS-CoV-2 viruses. This new dataset was processed using the already trained SVM and MLP models, as shown in Figure 3.

### 2.6. Dataset Distinguishability Analysis

Nowadays, data visualization approaches can help us understand the distribution of a dataset and intuitively investigate whether a dataset is distinguishable or not. T-distributed stochastic neighbor embedding (t-SNE) is a tool to visualize high-dimensional data. It converts similarities between data points to joint probabilities and tries to minimize the Kullback–Leibler divergence [50] between the joint probabilities of the low-dimensional embedding and the high-dimensional data.

We implemented the t-SNE tool on the specimen detection datasets to interpret the distinguishability of the datasets. The data distribution patterns could help interpret the performance of the models on the dataset. Both the raw dataset and the features extracted from the raw data were considered in terms of their distinguishability. We also investigated whether the extracted features had distributions different from those of the raw data.

## 3. Results and Discussion

In terms of the experiments, we used SVM and MLP models to test the raw data processing and feature engineering method. Two performance metrics were considered in the experiments, sensitivity (SEN) and specificity (SPE), which are defined as

(1) $$SEN=\frac{TP}{TP+FN}$$

(2) $$SPE=\frac{TN}{TN+FP}$$

where TP, FN, TN, and FP stand for true positive, false negative, true negative, and false positive, respectively.

Table 1 shows the performance of the ML model predictions. We can see from the last two rows that perfect performance was achieved for both the raw data and the feature engineering methods, combined with either the SVM or MLP model. The fourth and fifth rows in Table 1 show the performance of the models in processing the control experiment dataset. The performance was very poor, and this was due to the fact that the biosensors had not been functionalized with specific antibodies and, thus, could not detect the SARS-CoV-2 virus effectively. The low *p*-values for both the control detection and valid detection datasets demonstrate the reliability of the classification of the two types of sample datasets.

**Figure 4:**
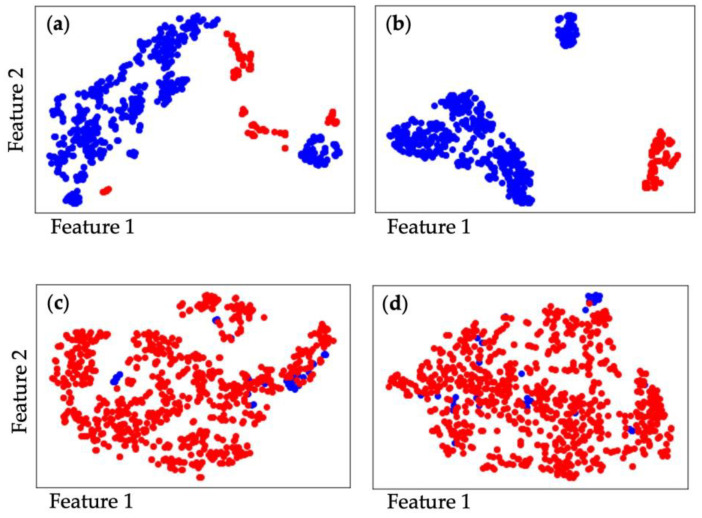
The t-SNE data visualization results of experimental SARS-CoV-2 detection dataset; red and blue represent the positive and negative samples, respectively. (**a**) Raw dataset of valid detection experiment; (**b**) feature engineering dataset of valid detection experiment; (**c**) raw dataset of the control detection experiment; (**d**) feature engineering dataset of control detection experiment.

Figure 4a shows the data distribution of the raw datasets in 2D space with the t-SNE data visualization approach. We can see that the positive and negative samples from the dataset of the valid detection experiments are clustered without any overlapping. Thus, the valid experimental dataset is distinguishable. Figure 4b shows the data distribution of the features extracted from the dataset in Figure 4a. The extracted features changed the data distribution while maintaining distinguishability because the samples were separated into different clusters. Figure 4c shows the data distribution of the datasets obtained from the control experiments wherein biosensors were not functionalized with specific antibodies. Negative samples overlap the positive samples, and the dataset is indistinguishable according to the visualization results. Figure 4d shows the data distribution of the features extracted from the dataset in Figure 4c. The distribution of the features’ dataset is still mixed up, so that feature engineering cannot help the dataset to be classified effectively. These dataset distribution results can serve to interpret the performance comparisons demonstrated in Table 1.

Table 2 demonstrates the advantages of the ML data processing technique compared with other techniques. The general advantages of ML are valid, in addition to the eased hardware requirement.

To verify the efficacy of the ML data processing technique for biosensors, detection experiments of inactivated SARS-CoV-2 at the vaccination sites of the Hangzhou Center for Disease Control and Prevention (CDC) were carried out, and the detection results were compared with the gold standard: reverse-transcription qPCR technique. The environmental specimens were collected from various locations at different vaccination sites, delivered to Hangzhou CDC within 4 h, and were simultaneously analyzed using both techniques. Table 3 shows that the biosensors, together with the ML data processing, generated detection results that were consistent with the qPCR results. Note that qPCR provides semiquantitative results dependent on the Ct value [5], while the ML processing of biosensor data only provides qualitative results. This comparative study demonstrates that the ML technique is an effective tool for biosensor signal and data processing,

## 4. Conclusions

In this work, machine learning techniques were used to process the signals and datasets of photonic biosensors. Both SVM and MLP were used to process raw data and future engineering data, and perfect results were obtained that distinguished between negative and positive detections. Control experiments were also carried out, wherein biosensors not functionalized with specific antibodies were used to detect SARS-CoV-2 virus. Both the SVM and MLP models trained with valid experimental data could not distinguish between the negative and positive detections in the control experiments. To demonstrate the distinguishability of the raw data and the future engineering data for both valid experiments and control experiments, we implemented a t-SNE data visualization approach. The results showed that the valid experimental dataset was distinguishable, and the control experimental dataset was indistinguishable according to both the raw data and the feature engineering methods. The results were consistent with the data processing performance of machine learning techniques achieved for the valid experimental dataset and the control experimental dataset. Future research will focus on ML techniques for the determination of quantitative detection results so that the quantity of target biospecies in specimens can be obtained. ML can be a powerful tool in processing the signals and datasets of biosensors for which there are salient features in the response signals of such biosensors. This includes optical, electrochemical, thermal, and mechanical biosensors.

## Figures and Tables

**Table 1:** Performance of raw data and feature engineering processing methods with two machine learning models.

Method	Raw Data						Feature Engineering					
**Model**	SVM			MLP			SVM			MLP		
**Parameter**	SEN	SPE	AUC	SEN	SPE	AUC	SEN	SPE	AUC	SEN	SPE	AUC
**Performance on** **Control Detection Data**	100%	46%	60.1%	86%	46%	63.4%	29%	33%	38.8%	27%	32%	38.1%
***p*-Value for Control Detection Data**	<0.05											
**Performance on Valid Detection Data**	100%											
***p*-Value for Valid Detection Data**	<0.05											

SVM: support vector machine; MLP: multilayer perceptron; SEN: sensitivity; SPE: specificity; AUC: area under ROC curve; ROC: receiver operating characteristic.

**Table 2:** Comparison of machine learning techniques with other signal processing techniques.

	Factor	Need Data Filtering and Denoising	Need to Take Care of Shift Direction	Need Stable Light Source and Low Noise Spectroscopy System	Needed Researcher Work
Technique					
Find peaks and calculate spectral shift		Yes	Yes	No	Algorithm design and test
Interferogram average over wavelength		Yes	No	Yes	Algorithm design and test
Intensity interrogation		Yes	No	Yes	Algorithm design and test
Machine learning		Yes	No	No	Model training from data

**Table 3:** Comparison of detection results of inactivated SARS-CoV-2 at vaccination sites of Hangzhou CDC using both qPCR technique and biosensor with ML technique.

Specimen Collection Location		qPCR Result	Biosensor with ML Result
Vaccination Site 1	Operation Desktop	Weak positive	Positive
Vaccination Site 1	Vaccination Station	Strong positive	Positive
Vaccination Site 2	Operation Desktop	Weak positive	Positive
Vaccination Site 2	Vaccination Station	Weak positive	Positive
Vaccination Site 2	Ventilation Plate	Strong positive	Positive
Vaccination Site 2	Inoculation Table Handle	Weak positive	Positive
Vaccination Site 4	Keyboard and Mouse	Negative	Negative
Vaccination Site 5	Pen and White Board	Strong positive	Positive
Vaccination Site 55	Inoculation Table Handle	Negative	Negative
No. 4 and No. 5 Inoculation Desk Room	Door Handle and Switch	Negative	Negative
Other	Hemostatic Swab	Weak positive	Positive
Other	Cleaner’s Hand	Negative	Negative

## Data Availability

The data presented in this study are available on request from the corresponding author. The data are not publicly available due to privacy.
